# Fe(III)-Based Nanomicelles for Magnetic Resonance Imaging of Colorectal Cancer with Hepatic Metastasis

**DOI:** 10.3390/jfb16070229

**Published:** 2025-06-20

**Authors:** Tianlun Shen, Kaiwei Lv, Zhenyan Chen, Songyi Xu, Guangyao Li, Guocan Han, Yì Xiáng J. Wáng, Jun Ling, Jihong Sun

**Affiliations:** 1 Department of Radiology, Sir Run Run Shaw Hospital, School of Medicine, Zhejiang University, Hangzhou 310016, China; tlshen@zju.edu.cn (T.S.); 22318150@zju.edu.cn (K.L.); 8014033@zju.edu.cn (Z.C.); ligy_19@126.com (G.L.); hanguocan@zju.edu.cn (G.H.); lingjun@zju.edu.cn (J.L.); 2 MOE Key Laboratory of Macromolecular Synthesis and Functionalisation, Department of Polymer Science and Engineering, Zhejiang University, Hangzhou 310058, China; xusongyi@zju.edu.cn; 3 Department of Imaging and Interventional Radiology, Faculty of Medicine, The Chinese University of Hong Kong, Shatin, Hong Kong SAR 999077, China; yixiang_wang@cuhk.edu.hk; 4 Key Laboratory for Biomedical Engineering of Ministry of Education, Zhejiang University, Hangzhou 310027, China; 5 Cancer Center, Zhejiang University, Hangzhou 310058, China

**Keywords:** poly(α-amino acid)s, nanomicelles, magnetic resonance imaging, colorectal cancer, hepatic metastasis

## Abstract

Colorectal cancer (CRC) is a leading global malignancy with a poor prognosis in advanced stages. Early and accurate diagnosis remains challenging due to the overlapping of clinical manifestations between early-stage CRC and inflammatory bowel diseases. Although dynamic contrast-enhanced MRI (DCE-MRI) is a critical imaging modality for the diagnosis of CRC and colorectal cancer liver metastasis (CRLM), conventional gadolinium-based contrast agents (GBCAs) have the limitations of rapid clearance and potential toxicity risks. In this study, we report a gadolinium-free T1-weighted nanocontrast agent based on Fe(III)-coordinated poly(α-amino acid)s (Fe@POS) nanomicelles. Fe@POS nanomicelles exhibit a high longitudinal relaxivity (r_1_ = 5.56 mM^−1^s^−1^) and prolonged blood circulation time with selective CRC tumor accumulation via enhanced permeability and retention (EPR) effect. In vivo MRI studies revealed long-period MRI of CRC. In CRLM lesions, normal hepatic tissue demonstrates greater Fe@POS uptake compared to tumor tissue, which enables clear delineation of tumor margins on MRI. Histological and biochemical analysis confirmed the biocompatibility of Fe@POS nanomicelles, with no acute toxicity observed, highlighting their potential as alternatives to GBCAs for clinical diagnostic applications.

## 1. Introduction

Colorectal cancer (CRC) is the third most prevalent cancer worldwide [1,2], with a steady rise in incidence in recent years [3]. The cancer stage at which CRC is diagnosed significantly impacts patient survival rates. Patients with Stage I CRC exhibit a five-year survival probability of 90%, whereas the probability dramatically declines to 14% in Stage IV CRC cases with distant metastasis [4]. However, CRC patients present similar clinical manifestations with inflammatory bowel diseases during Stage I and Stage II, resulting in significant diagnostic difficulties [5]. Furthermore, 20–35% of CRC patients develop hepatic metastasis when diagnosed (Stage IV). Current clinical evidence confirms that hepatic metastasectomy significantly improves five-year survival probability in patients with colorectal cancer liver metastasis (CRLM) [6], thereby underscoring the critical importance of diagnosing and localizing hepatic metastasis in advanced CRC patients.

Clinically, as a non-invasive imaging technique, dynamic contrast-enhanced magnetic resonance imaging (DCE-MRI) has become the cornerstone in the diagnosis of CRC and CRLM, offering high-resolution soft tissue contrast while eliminating ionizing radiation risks [7,8]. Gadolinium-based contrast agents (GBCAs), primarily gadopentetate dimeglumine (Gd-DTPA) and gadolinium tetra-azacyclododecane tetraacetic acid (Gd-DOTA), currently constitute the majority of clinical T1-weighted MRI contrast agents. Nevertheless, the small molecular GBCAs lead to rapid vascular extravasation from the blood capillaries into tissue and a suboptimal imaging window, which hampers the detection of minute primary CRC and CRLM lesions [9]. More critically, Gd(III) complexes based on linear ligands may lead to Gd(III) ion leakage, potentially causing nephrogenic systemic fibrosis (NSF) in renal-impaired patients and exhibit long-term tissue accumulation [10,11,12]. Accordingly, these limitations warrant developing more biocompatible and effective alternatives to GBCAs to achieve precise DCE-MRI of CRC and CRLM.

Fe(III) ions possess five unpaired electrons in their 3d orbital, exhibiting a paramagnetic property, which enables their application as MR contrast agents [13,14]. On the other hand, as an essential trace element in the human body, Fe(III) shows superior biosafety and biocompatibility compared to Gd(III) [15]. Various Fe(III)-based medicines have been widely applied in iron-deficiency anemia, including Ferric Derisomaltose, Ferric Carboxymaltose and Ferrous Fumarate [16,17,18]. Currently, the superparamagnetic iron oxide formulation Ferumoxytol is approved by the U.S. Food and Drug Administration for the magnetic resonance angiography of the aorta in patients with acute myocardial infarction [19]. For Fe(III)-coordinated composite nanoparticles, a number of studies have achieved enhanced longitudinal relaxivity (>5.0 mM^−1^s^−1^) by accelerating the inner-sphere water exchange rate [20,21,22]. The blood circulation time of Fe(III)-coordinated nanoparticles can be prolonged through appropriate hydrophilic surface modification, thus contributing to the long-duration DCE-MRI [23,24,25].

Compared to small-molecular GBCAs, properly engineered nanoparticles can accumulate in CRC lesions through the enhanced permeability and retention (EPR) effect, thereby enabling tumor-specific imaging [26,27]. These tumor-specific MR nanocontrast agents facilitate the precise diagnosis of CRC and its hepatic metastasis [28]. Poly(α-amino acid)s (PAAs), a class of protein-mimetic polymers including polypeptides and polypeptoids, exhibit exceptional biocompatibility and biodegradability [29]. These polymers can spontaneously self-assemble into ordered nanostructures, with the resulting assemblies capable of encapsulating therapeutic agents or imaging probes [30,31]. Their structural similarity to native proteins enables diverse biomedical applications, including stimuli-responsive biomaterials, controlled drug delivery and biomedical imaging [32,33,34,35]. Based on the above-mentioned findings, the polymer-Fe(III) coordinated nanoparticles with high hydrophilicity, weak serum protein adsorption and prolonged blood circulation are promising alternatives to GBCAs, promoting the effective DCE-MRI of CRC with hepatic metastasis.

**Figure 1:**
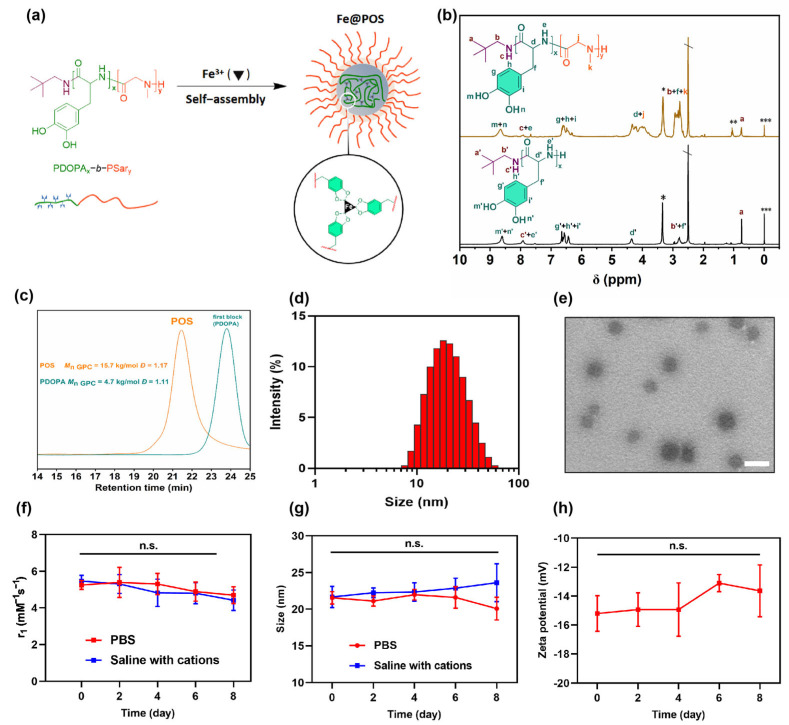
Physiochemical characterization of POS and Fe@POS. (**a**) Schematic illustration of self-assembly of Fe@POS nanomicelles. (**b**) ^1^H NMR spectrum of POS in deuterated dimethyl sulfoxide (DMSO-*d*_6_) (*: H_2_O, **: Ether, ***: Tetramethyl silane, \ DMSO). (**c**) SEC traces (5 wt‰ in hexafluoroisopropanol) of POS and first block of POS(PDOPA_21_). (**d**) The size distribution of Fe@POS nanomicelles analyzed by dynamic light scattering. (**e**) Transmission electron microscope images of Fe@POS nanomicelles. Scale bar: 50 nm. The longitudinal relaxivity r_1_ (**f**) and size (**g**) variation of Fe@POS nanoprobes in phosphate buffer solution (PBS) or saline with endogenous cations (15 μmol/L Zn^2+^, 1 mmol/L Ca^2+^, 1 mmol/L Mg^2+^) within 8 days (pH = 7.4). (**h**) The zeta potential variation of Fe@POS nanoprobes in deionized water over 8 days. n.s.: not significant.

The purpose of this study is to develop a gadolinium-free T1-weighted contrast agent for long-period MRI of CRC and CRLM. We herein report a Fe(III)-polymer-coordinated nanomicelles (Fe@POS) by self-assembling Fe(III) and poly(3,4-dihydroxy-L-phenylalanine)-*b*-polysarcosine (PDOPA-*b*-PSar, abbr. POS) diblock copolymer (Figure 1a). The catechol moieties on PDOPA block chelate and anchor Fe(III) into the core of nanomicelles to achieve T1-weighted MRI. In vivo studies on the CRC patient-derived tumor xenograft model and CRC hepatic metastasis model demonstrate that Fe@POS nanocontrast agents exhibit long-period MRI of CRC. In CRLM lesions, the differential uptake kinetics of Fe@POS between normal hepatic tissues and CRLM delineate metastasis margins. Furthermore, we evaluated the biocompatibility of Fe@POS nanomicelles to elucidate their potential clinical application.

## 2. Materials and Methods

### 2.1. Materials

Sarcosine, 3,4-dihydroxy-L-phenylalanine (L-DOPA), phenyl chloroformate (PCF), neopentylamine (NPA), hexafluoroisopropanol (HFIP), *N*,*N*-diisopropylethylamine (DiPEA), potassium trifluoroacetate (CF_3_COOK), *N*,*N*-dimethylacetamide (DMAc) and deuterated dimethyl sulfoxide (DMSO-*d*_6_) were purchased from Energy Chemical (Shanghai, China). Diethyl ether, petroleum ether (PE), fluorescein isothiocyanate isomer I (FITC), dimethyl sulfoxide (DMSO), ferric nitrate nonahydrate (Fe(NO_3_)_3_·9H_2_O) and sodium ethoxide (EtONa) were purchased from Sinopharm Chemical Reagent Co., Ltd. (Shanghai, China). Gd-DTPA was purchased from Beilu Pharmaceutical Co., Ltd. (Beijing, China). Neopentylamine was stirred over CaH_2_ and then followed by distillation under reduced pressure. Other materials were used as received.

### 2.2. Characterization

Nuclear magnetic resonance spectra (NMR) were recorded on a Bruker Avance DMX 400 spectrometer (^1^H: 400 MHz, Bruker, Germany). The size exclusion chromatography (SEC) instrument consisted of a Waters 1515 isocratic HPLC pump (Waters, MA, USA), a Waters 2414 interferometric refractometer (RI, Waters, MA, USA) and two Shodex KF (Resonac Corporation, Tokyo, Japan) series columns. HFIP containing 3 mg/mL CF_3_COOK was used as the eluent with a flow rate of 0.8 mL/min at 40 °C. Commercial poly (methyl methacrylate) standards (Agilent, Santa Clara, CA, USA) with narrow dispersity were used for molecular weight (MW) calibration. The concentrations of Fe(III) were measured by inductively coupled plasma mass spectrometry (ICP-MS) on a Thermo XSeries II spectrometer (Thermo Fisher Scientific, Waltham, MA, USA). Dynamic light scattering (DLS) measurements were carried out using a particle size analyzer (Zetasizer Nano Series, Malvern Instruments, Malvern, UK) at 25 °C. Transmission electron microscope (TEM) measurements were carried out using a JEM-1400plus electron microscope (Japan Electron Optics Laboratory Co., Ltd., Akishima, Japan).

### 2.3. Synthesis of N-Phenoxycarbonyl-sarcosine (Sar-NPC) and N-Phenoxycarbonyl-3,4-dihydroxy-L-phenylalanine (DOPA-NPC)

A suspension of sarcosine (22.27 g, 0.25 mol) and PCF (31.1 mL, 0.25 mol) in 250 mL of EA was heated at 45 °C for 48 h. The reaction mixture was filtered and evaporated under reduced pressure to obtain a crude product, which was purified by chromatography (a gradient eluent EA:PE = 1:5–1:1) to obtain Sar-NPC as a light-yellow viscous liquid (11.67 g, yield: 22.5%).

A suspension of L-DOPA (29.56 g, 0.15 mol) and PCF (19.1 mL, 0.15 mol) in 250 mL of EA was heated at 45 °C for 48 h. The reaction mixture was filtered and evaporated under reduced pressure to obtain a crude product, which was purified by chromatography (a gradient eluent EA:PE = 1:5–1:1) to obtain an EA solution of L-DOPA-NPC. L-DOPA-NPC, as a white powder, was obtained after the evaporation of EA (8.35 g, yield: 17.4%).

### 2.4. Diblock Copolymerization of DOPA-NPC with Sar-NPC

Diblock copolymerization was performed using the Schlenk technique and all vials were flame-dried and purged with argon. L-DOPA-NPC (0.4050 g, 1.276 mmol) was dissolved in 1.30 mL of DMAc, followed by 1.27 mL of neopentylamine in DMAc solution (0.0956 mol/L). The vial was sealed and placed in a 60 °C oil bath for 24 h. A total of 2.00 mL of the first block solution was transferred to a solution of Sar-NPC (3.016 g, 14.42 mmol) and DiPEA (0.3044 g, 2.336 mmol) dissolved in 28.0 mL dry DMAc. The vial was sealed and placed in a 60 °C oil bath for 48 h. The diblock copolymer POS was precipitated from diethyl ether and dried under vacuum (POS, 0.598 g, yield: 50%).

### 2.5. Preparation of Fe@POS Nanomicelles

Preparation of Fe@POS nanomicelles was performed using the Schlenk technique and all vials were purged with argon. POS (155 mg, 0.151 mmol of DOPA unit) was dissolved in 3.0 mL DMSO. Then, 3.0 mL DMSO solution of EtONa (10.8 mg, 0.158 mmol) and 3.0 mL DMSO solution of Fe(NO_3_)_3_·9H_2_O (62.7 mg, 0.155 mmol) were added with continuous stirring. After 30 min, deionized water (20 mL after argon bubbling to remove oxygen) was slowly added into the solution using the Perfusor^®^ (Annuo Medical Device Co., Ltd., Ningbo, China) compact in 6 h. The mixture was dialyzed against deionized water for 24 h by changing the dialysis medium every 8 h to remove impurities. The Fe@POS nanomicelles were concentrated using ultrafiltration centrifugal technology. When the concentration of POS in Fe@POS is 1.0 mg/mL, the concentration of Fe(III) is found to be 16.1 μg/mL according to ICP-MS analysis. For the FITC-labeled Fe@POS, 2.0 mg FITC was added to the reaction of POS and Fe(NO_3_)_3_·9H_2_O, and the dialysis process was carried out under dark conditions.

### 2.6. Cell Culture and Animal Models

The mouse-derived colon carcinoma (CT26) cells were cultured in Roswell Park Memorial Institute 1640 medium (Gibco, Thermo Fisher Scientific, Waltham, MA, USA), supplemented with 10% fetal bovine serum and 1% penicillin/streptomycin. The Madin–Darby canine kidney (MDCK) cells, human colon adenocarcinoma (Caco-2) cells and human hepatocellular carcinoma (HepG2) cells were cultured in high-glucose Dulbecco’s modified Eagle’s medium (Gibco), supplemented with 10% fetal bovine serum and 1% penicillin/streptomycin. All cells were cultivated at 37 °C in a 5% CO_2_ atmosphere using a cell-humidified incubator (Thermo Fisher Scientific, Waltham, MA, USA).

Animal experiments followed the protocols and standards for the care and use of experimental animals according to the local animal welfare committee (Hangzhou Province, China; approval number: SRRSH202310001). For the CT26 subcutaneous xenograft tumor model, female BALB/c mice (6 weeks old) were subcutaneously inoculated with 1 × 10^6^ CT26 cells in the right inguinal region. Tumor volumes were measured every 3 days using calipers, and calculated as follows:

Tumor volume = (length × width^2^)/2 (1)

Mice were maintained under specific pathogen-free conditions and humanely euthanized when tumors reached 1500 mm^3^. Patient-derived xenograft (PDX) models were established by subcutaneously implanting fresh tumor fragments (3 mm^3^) from surgical specimens into 6-week-old immunodeficient BALB/c nude mice. Tumor tissues were processed within 4 h post-resection in sterile PBS, and the tumor fragments were engrafted in the right dorsal region of mice. All procedures were performed under isoflurane anesthesia in a laminar flow hood. The CRLM models were established in 6-week-old BALB/c mice. Under isoflurane anesthesia, a 1-cm left flank incision was made to expose the spleen. A suspension of 10^5^ CT26 tumor cells in 50 μL PBS was slowly injected into the inferior splenic pole using an insulin syringe (Becton, Dickinson and Company, Franklin Lakes, NJ, USA). After clamping for 3 min to prevent leakage, the spleen was returned to the abdominal cavity. The abdominal wall and skin were sutured separately. Mice were monitored daily for 3 days post-operation and every 3 days thereafter. Liver metastasis was quantified by bioluminescent imaging at 2 weeks post-injection.

### 2.7. MRI Analysis

Magnetic resonance scanning was performed on a 3.0 T MR scanner (SignaHDxt, GE Medical Systems, Chicago, IL, USA) using a special small animal coil (Shanghai Chenguang Medical Technologies Co., Ltd., Shanghai, China). Longitudinal relaxivity r_1_ was obtained by an inversion recovery pulse sequence with variable inversion times. Longitudinal relaxivity r_1_ of Fe@POS was measured at 20 °C room temperature. Briefly, Fe@POS was dissolved in distilled water and diluted in a series of concentration gradients ([Fe] = 0.25, 0.20, 0.15, 0.10, 0.05 mM) before being encapsulated into 200 μL tubes. Inversion times were set as 50, 100, 200, 300, 400 and 600 milliseconds for all samples. The constant r_1_ was obtained by linearly fitting the reciprocal of T1-time to concentration. For the in vivo MR imaging, A T1-weighted spin-echo sequence was used with the following parameters: TR/TE = 500/100 ms, matrix = 256 × 256, slice thickness = 2 mm, FOV = 11 × 11 cm^2^, Nex = 2. The CT26 subcutaneous tumor model, the CRC-PDX model and the CRC-CRLM model were used in the in vivo MR imaging. All mice were scanned after isoflurane anesthesia and then intravenously injected with 200 μL Fe@POS (0.1 mmol Fe/kg), followed by scanning at different time points (5 min, 1 h, 2 h, 3 h, 4 h, 6 h, 8 h and 24 h). As a control, Gd-DTPA (0.1 mmol Gd/kg) was tested on the CT26 subcutaneous tumor model using the same MR scanning parameters.

The signal-to-noise ratio (SNR) values were calculated as follows:

SNR = SI_ROI_/SD_background_ (2)

where SI_ROI_ is the signal intensity of the region of interest, SD_background_ is the standard deviation of the background signal from non-tissue regions.

### 2.8. Biodistribution and Histological Analysis

Female BALB/c mice bearing CT26 subcutaneous tumors were randomly divided into three experimental groups and one control group (*n* = 3). The mice of the experimental group were intravenously administered with 200 μL Fe@POS (0.1 mmol Fe/kg) solution, whereas the mice of the control group were intravenously injected with an equal volume of PBS. Then, the mice of the experimental groups were euthanized with ether at 12, 24 and 48 h time points, subsequently followed by the dissection and collection of major organs (heart, lung, liver, spleen, kidney and tumor). Organ samples were weighed and predigested in 6.0 mL concentrated nitric acid for 48 h and digested at 250 °C for 1 h. The remaining clear solution (about 0.5 mL) was diluted to 6.0 mL with 1% HNO_3_ and the Fe concentration was quantified by ICP-MS. For the histological analysis, organ and tumor sections were stained with hematoxylin and eosin according to standard protocol and observed under an optical microscope.

## 3. Results and Discussion

### 3.1. Synthesis and Characterization of Fe@POS Nanomicelles

The synthesis route of amphiphilic diblock PDOPA-*b*-PSar copolymers is detailed in Supplementary Figures S1 and S2. First, the monomers DOPA-NPC and Sar-NPC were synthesized through the carbamoylation of DOPA and sarcosine with phenyl chloroformate, respectively (Scheme S1). Their structures were confirmed by ^1^H NMR (Figures S1 and S2). Subsequently, PDOPA-*b*-PSar copolymers were synthesized via neopentylamine-initiated sequential polymerization of DOPA-NPC with Sar-NPC (Scheme S2). The ^1^H NMR spectrum of POS elucidated the complete assignment of the proton signals for both DOPA and sarcosine repeating units (Figure 1b and Figure S3). The signal of methyl protons of sarcosine units (H^k^) and methylene protons of DOPA *β*-carbons (H^f^) and neopentylamine residues (H^b^) appears in the broad range of 2.55–2.97 ppm. The characteristic signals of DOPA units observed at 8.54–8.92 ppm (H^m, n^), 7.51–8.00 ppm (H^e^) and 6.38–6.81 ppm (H^g, h, i^) were attributed to the phenolic hydroxyl, amide and aromatic protons, respectively. According to the signal intensities of terminal tert-butyl group protons (H^a^) and the characteristic signals of sarcosine units (H^k^) and DOPA units (H^g, h, i^), the composition of the copolymer was calculated as PDOPA_21_-*b*-PSar_120_ and the molecular weight was 12.4 kg/mol. SEC traces revealed that the retention time of POS decreased from the first block of PDOPA, suggesting that all of the terminal amine groups of PDOPA successfully initiated the polymerization of Sar-NPC (Figure 1c).

The amphiphilic PDOPA-*b*-PSar copolymer was self-assembled into polymeric micelles in the aqueous phase with the PDOPA block chelating Fe(III) through catechol coordination (Figure 1a). The Fe(III)-loaded nanomicelles were abbreviated as Fe@POS. The hydrophobic PDOPA block, along with the chelated paramagnetic Fe(III) ions, is confined in the core of the Fe@POS nanomicelles, while the hydrophilic PSar block remains in direct contact with the aqueous solvent. Quantitative ICP-MS analysis revealed an Fe(III) encapsulation efficiency of 27.94 ± 4.14% and a loading capacity of 1.20 ± 0.17%, respectively. DLS results suggested a hydrodynamic size of 20.4 nm and a low polydispersity index (PDI) of 0.208 (Figure 1d), indicating monodisperse micelles. TEM imaging confirmed that the Fe@POS nanomicelles were spherical with a uniform diameter of 28 ± 4 nm (Figure 1e). The nanoprobes maintained colloidal stability in phosphate buffer solution (PBS) and saline containing endogenous cations (Zn^2+^, Ca^2+^, Mg^2+^), without significant changes in particle size and longitudinal relaxivity over 8 days (Figure 1f,g). The zeta potential of Fe@POS is −15.2 ± 0.3 mV and remained stable over 8 days (Figure 1h). The successful preparation of size-stable Fe@POS nanomicelles suggests potential application for in vivo MRI.

### 3.2. In Vitro and In Vivo T1-Weighted MRI in CRC and CRLM Models

**Figure 2:**
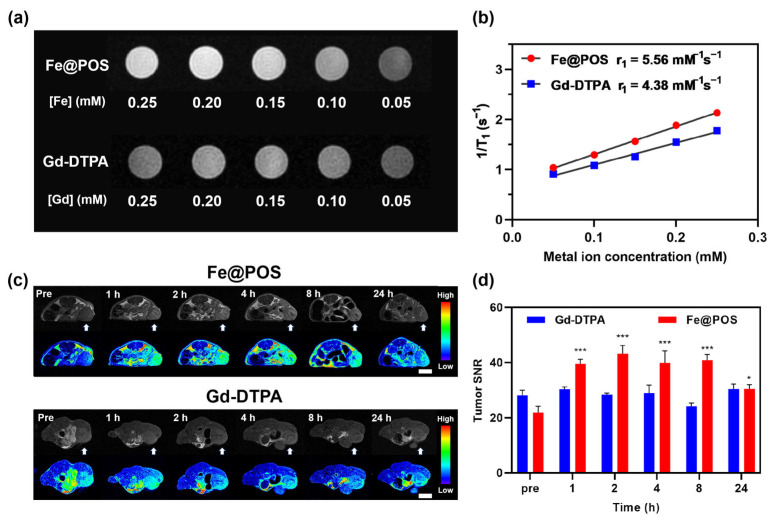
In vitro and in vivo MRI study of Fe@POS and Gd-DTPA on CT26 tumor-bearing mice. (**a**) T1-weighted images of Fe@POS and Gd-DTPA at different Fe/Gd concentrations. (**b**) Linear fitting of the reciprocal of T1 relaxation time to metal ion concentrations. (**c**) T1-weighted MR images at different times of CT26 tumor-bearing mice treated with Fe@POS and Gd-DTPA. White arrow: tumor region. In pseudo-color images, blue to red represents low to high T1 signal intensity. Scale bar: 1 cm. (**d**) Signal-to-noise ratio change of tumor region in (**c**). * *p* < 0.05, *** *p* < 0.001 were considered statistically significant.

In vitro T1-weighted MRI demonstrated concentration-dependent signal enhancement (Figure 2a) with Fe@POS achieving an r_1_ of 5.56 mM^−1^s^−1^ (Figure 2b), which is 1.3-fold higher than the clinical standard Gd-DTPA (4.38 mM^−1^s^−1^). Analogous to GBCAs, the proton relaxivity enhancement of Fe(III)-coordinated complexes is primarily mediated through inner-sphere, second-sphere and outer-sphere mechanisms [15,36]. The contribution of the inner-sphere mechanism is directly proportional to the number of water molecules that directly coordinate with the central Fe(III) ions, where the hydration state of Fe(III) ions affects the T1 signal [15,36]. However, literature reports indicate that for Fe(III)-catecholate coordinated complexes, the Fe(III) coordination sites are predominantly occupied by tris-catecholate ligands, preventing the hydration of Fe(III) with inner-sphere water molecules [21,37,38]. Therefore, we speculated that the inner-sphere mechanism contributes negligibly to the T1 relaxation capability, while the second-sphere and outer-sphere mechanisms mainly govern the T1 relaxation of the Fe@POS nanoprobes. It is worth noting that the catecholate groups coordinated with Fe(III) ions in Fe@POS can form extensive hydrogen bonds with water molecules, which significantly enhances the interaction between Fe(III) ions and second-sphere water molecules. This effect promotes the contribution of the second-sphere mechanism in Fe(III)-catecholate coordinated complexes [21,39]. Besides, the outer-sphere water molecules diffusing close to Fe(III) centers also undergo relaxation [40]. Given the enriched Fe(III) ions in Fe@POS, the outer-sphere mechanism is expected to contribute to the T1 relaxation.

In vivo T1-weighted MRI was performed on a 3.0 T MRI system using the CT26 subcutaneous tumor model and CRC patient-derived tumor xenograft (PDX) models. Transverse MR images of CT26-tumor-bearing mice intravenously administered with Fe@POS and Gd-DTPA are shown in Figure 2c,d. MR signal intensities of tumor regions were remarkably enhanced 1 h post-Fe@POS injection (0.1 mmol Fe/kg) and remained elevated for 8 h. The tumor signal-to-noise ratio (SNR) has a 2.0-fold increase compared to the baseline scan with a statistically significant difference (Figure 2d, *p* < 0.001). Moreover, the signal enhancement persisted after 24 h, indicating the prolonged tumor retention properties of Fe@POS. Research has reported that nanoparticles with a size range of 20–70 nm tend to penetrate solid tumors via the EPR effect while avoiding rapid clearance by the kidneys and macrophages in the liver and spleen [41,42]. The optimal particle size of Fe@POS nanoprobes facilitates their prolonged accumulation in tumors through passive targeting. For comparison, clinically applied Gd-DTPA was used as a control to assess the in vivo MRI performance of Fe@POS. Mice intravenously injected with Gd-DTPA (0.1 mmol Gd/kg) displayed minimal enhancement in the tumor region during 24 h (Figure 2c,d).

**Figure 3:**
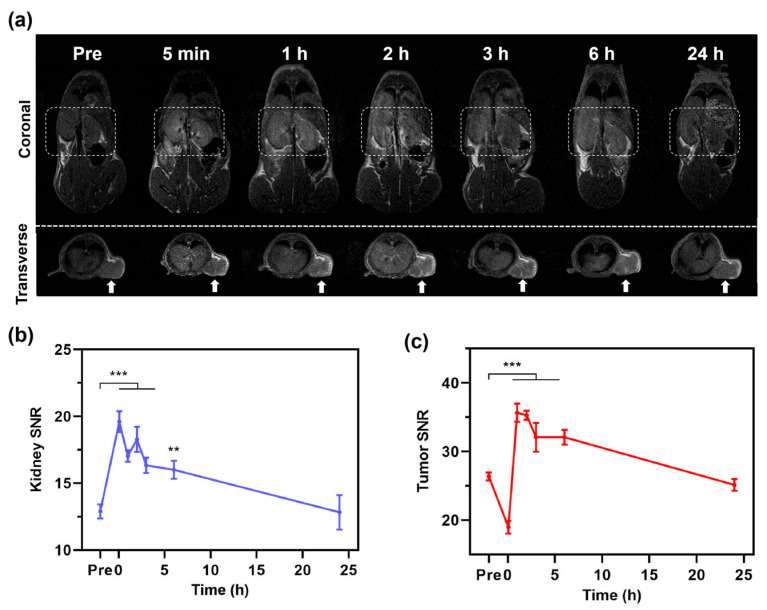
In vivo MRI study of Fe@POS using CRC patient-derived xenograft tumor models. (**a**) T1-weighted MR images at different times of CRC-PDX mice treated with Fe@POS. White box: kidney region. White arrow: tumor region. (**b**,**c**) Signal-to-noise ratio change of kidney (**b**) and tumor (**c**). ** *p* < 0.01, *** *p* < 0.001 were considered statistically significant.

The CRC-PDX models retain the molecular and histopathological characteristics of the primary tumor from patients, providing an accurate recapitulation of tumor microenvironment and heterogeneity [43]. During the MR imaging in CRC-PDX models, coronal MR imaging revealed rapid renal enhancement post Fe@POS injection with signal intensities increasing from 2.79 ± 0.13 to 4.34 ± 0.36, followed by a gradual decrease in the imaging process, which indicates gradual renal clearance of Fe@POS nanomicelles (Figure 3a,b). Similar to kidney enhancement, the tumor MR signal of transverse pictures peaked at 1 h before returning to baseline level by 24 h (Figure 3a,c). While Gd-DTPA is rapidly cleared from the body via the renal system (blood half-time < 30 min), the Fe@POS nanomicelles with high molecular weight and weak serum protein binding capacity prolong their blood circulation time, facilitating T1-weighted MRI tracking of CRC tumors.

**Figure 4:**
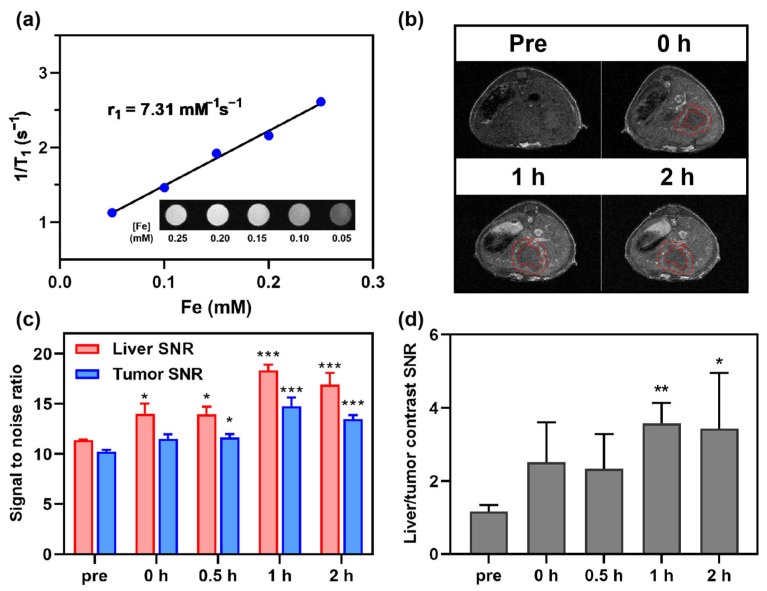
In vivo MRI study of Fe@POS using CRC colorectal cancer liver metastasis (CRLM) models. (**a**) The longitudinal relaxivity calculation of Fe@POS used in CT26-CRLM models. (**b**) T1-weighted MR images of CT26-CRLM mice. Red dashed line: delineated tumor margin. (**c**) Signal-to-noise ratio change of liver and tumor in (**b**). (**d**) Liver-tumor contrast signal-to-noise ratio at different times. * *p* < 0.05, ** *p* < 0.01, *** *p* < 0.001 were considered statistically significant.

The CRC-CRLM models were developed through splenic inoculation of CT26 colorectal tumor cells, allowing for hematogenous dissemination to the liver [44]. The high r_1_ Fe@POS nanomicelles were employed for in vivo MRI of CRC-CRLM mouse models (Figure 4a). Following intravenous administration of Fe@POS, the hepatic parenchyma exhibited rapid enhancement within 0.5 h, whereas tumor enhancement progressed gradually after 0.5 h (Figure 4b,c). Furthermore, the liver-to-tumor contrast SNR demonstrated progressive amplification from 1.16 ± 0.06 to 3.57 ± 0.61 (*p* < 0.01), significantly improving metastatic lesion detectability (Figure 4d). The contrast enhancement mechanism of Fe@POS nanoprobes in CRLM differs from that in CRC tumors. The liver is the primary barrier for the clearance of intravenously injected nanoparticles, with Kupffer cells considered to capture and accumulate the majority of nanoparticles in the liver [45,46,47]. Literature reports indicated that in liver metastasis-bearing models, the population size and phagocytic capacity of Kupffer cells in normal tissues are significantly higher than those in tumor regions [48]. Given that hepatic Kupffer cells exhibit pronounced phagocytic propensity for nanoparticles, a substantial proportion of intravenously administered Fe@POS nanoprobes underwent rapid hepatic uptake, manifesting as immediate and robust signal augmentation in normal liver parenchyma on MRI. In CRLM, Fe@POS accumulation occurred primarily through the EPR effect, culminating in peak tumoral contrast enhancement at approximately 1 h post-administration. Notably, while the absolute enhancement in CRLM lesions was lower in comparison to adjacent normal liver tissues, the differential enhancement kinetics between normal and metastatic tissues facilitated clear delineation of previously indistinct tumor margins (red dashed outlines, Figure 4b). This differential contrast pattern established an extended diagnostic window for CRLM detection. Currently, studies have reported the use of Mn(II)-doped silica nanoparticles and MnFe_2_O_4_ nanoparticles employing a similar differential contrast imaging modality for MRI of liver tumors [49,50].

### 3.3. Biosafety and Body Distribution of Fe@POS Nanomicelles

**Figure 5:**
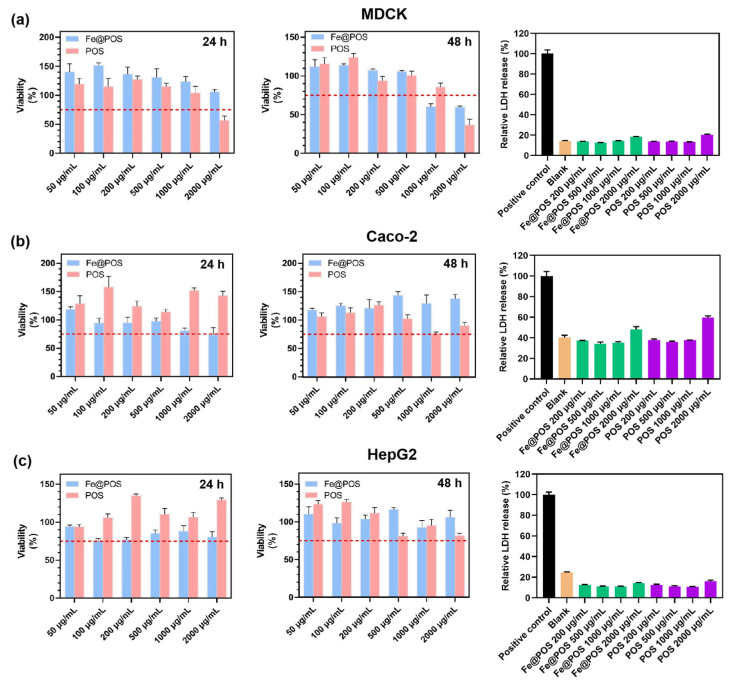
Effects of Fe@POS and POS on viability and membrane integrity in multiple cell lines. Cell viability of MDCK cells (**a**), Caco-2 cells (**b**) and HepG2 cells (**c**) co-cultured with POS and Fe@POS for 24 h (left) and 48 h (middle), the red dashed line represents the 75% cell viability. Relative release percentage of lactate dehydrogenase of MDCK cells (**a**), Caco-2 cells (**b**) and HepG2 cells (**c**) co-cultured with Fe@POS for 24 h (right). The positive control represents the maximum LDH release after cell lysis.

Given the long-term toxicity of gadolinium-based contrast agents [12,51], the biosafety of Fe@POS nanomicelles is crucial in clinical applications. The Fe@POS system comprises biocompatible and biodegradable poly(α-amino acid)-based polymers [52,53]. Additionally, Fe(III) was intentionally selected over Gd(III) as the T1-enhancing ion to eliminate risks associated with gadolinium leakage. In the comprehensive cell viability assays (Figure 5a–c), MDCK cells, Caco-2 cells and HepG2 cells maintained > 75% cell viability at a relatively high POS and Fe@POS concentration (1000 μg/mL). Cytotoxicity manifested only at suprapharmacological doses (>2000 μg/mL) on MDCK cells. Next, the cytotoxicity of Fe@POS nanomicelles was further assessed via lactate dehydrogenase (LDH) release assay, in which an elevated extracellular LDH level directly correlates with plasma membrane damage. Following 24 h co-culture with Fe@POS nanomicelles, MDCK cells, Caco-2 cells and HepG2 cells exhibited no significant plasma membrane damage compared to the positive control (Figure 5a–c).

**Figure 6:**
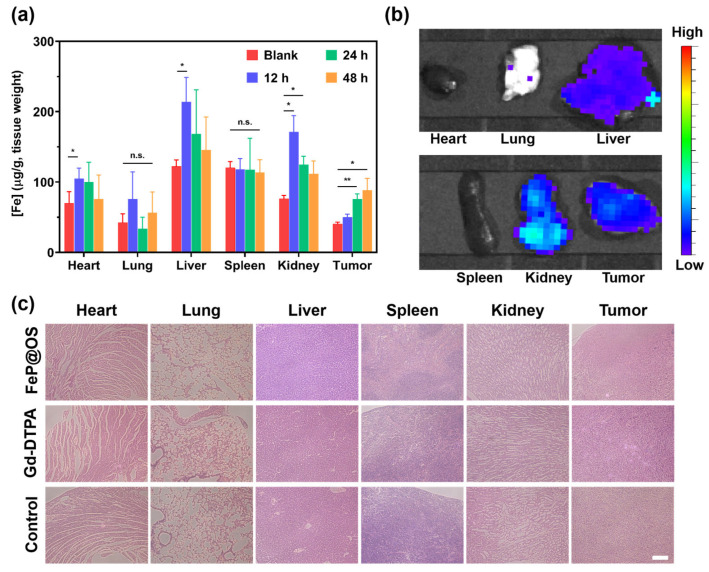
Biodistribution and histological analysis of mice treated with Fe@POS. (**a**) Biodistribution of Fe@POS in BALB/c mice after intravenous injection with Fe@POS. * *p* < 0.05, ** *p* < 0.01 were considered statistically significant. n.s.: not significant. (**b**) Ex vivo fluorescence images of tumor and major organs at 6 h post-Fe@POS injection. (**c**) Hematoxylin and eosin-stained tumor and major organ slices. Scale bar: 100 μm.

To systematically evaluate the in vivo behavior of Fe@POS nanomicelles, we performed biodistribution and histological analysis on blab/c mice. Quantitative ICP-MS revealed preferential accumulation of Fe@POS in the heart, liver, kidneys and tumor tissues with minimal pulmonary and renal uptake (Figure 6a). Ex vivo fluorescence imaging further confirmed the aggregation of Fe@POS in the liver, kidney and tumor regions (Figure 6b), which is consistent with the Fe distribution results. On the basis of the biological evaluation of medical devices, Part 11: Tests for systemic toxicity (ISO 10993-11:2017), we evaluated the acute systemic toxicity at 24 h [54]. Hematoxylin and eosin staining exhibited no significant histological abnormality in the major organs after Fe@POS injection (Figure 6c). All of the evidence collectively validates Fe@POS as a tumor-specific MRI nanocontrast agent with favorable biosafety and biocompatibility.

DCE-MRI is suggested as a sensitive imaging modality for the diagnosis of CRC and CRLM, but this potential is limited by the rapid in vivo clearance and non-tumor-specific biodistribution of GBCAs [55,56]. In this study, the Fe@POS nanoprobes demonstrate substantial potential by significantly prolonging the blood circulation time and enabling passive tumor targeting, facilitating high-sensitivity MRI of CRC and CRLM with an extended imaging time window. Consequently, the Fe@POS nanoprobes provide a distinct advantage in detecting primary and metastatic lesions. However, the absence of long-term biosafety evaluation in animal models represents a key limitation for the clinical application of Fe@POS nanoprobes. Future research will focus on systematic biosafety evaluation covering chronic and acute toxicity, immunogenic responses and potential carcinogenicity.

## 4. Conclusions

We have successfully developed Fe@POS nanomicelles as promising gadolinium-free MRI nanocontrast agents for CRC and CRLM diagnosis, addressing the limitations of conventional GBCAs. The Fe@POS nanomicelles demonstrate superior imaging performance with high longitudinal relaxivity (r_1_ = 5.56 mM^−1^s^−1^, 20 °C, 3.0 T MR scanner) and prolonged blood circulation, enabling an extended diagnostic window and improved CRC tumor detection. In the liver, the differential uptake of Fe@POS between normal hepatic tissues and CRLM delineates the margins of metastatic lesions. These findings provide a biocompatible nanoprobe as an alternative to GBCAs for clinical diagnostic applications.

## Data Availability

The original contributions presented in the study are included in the article/supplementary material; further inquiries can be directed to the corresponding author.

## Supplementary Materials

The following supporting information can be downloaded at https://www.mdpi.com/article/10.3390/jfb16070229/s1. Scheme S1. Synthesis of Sar-NPC (A) and L-DOPA-NPC (B). Scheme S2. Diblock copolymerization of L-DOPA-NPC with Sar-NPC. Figure S1. ^1^H NMR spectrum of Sar-NPC in DMSO-*d*_6_ (* H_2_O, ** DMSO). Figure S2. ^1^H NMR spectrum of L-DOPA-NPC in DMSO-*d*_6_ (* DMSO, ** TMS, \ diethyl ether). Figure S3. ^1^H NMR spectrum of POS in DMSO-*d*_6_ (* H_2_O, ** DMAc, *** Tetramethyl silane, \ DMSO).

## Abbreviations

The following abbreviations are used in this manuscript:

CRC	Colorectal cancer
DCE-MRI	Dynamic contrast-enhanced magnetic resonance imaging
CRLM	Colorectal cancer liver metastasis
GBCA	Gadolinium-based contrast agent
NSF	Nephrogenic systemic fibrosis
EPR	Enhanced permeability and retention
PAA	Poly(α-amino acid)
SNR	Signal-to-noise ratio
