# Supplementary material for: Fe(III)-Based Nanomicelles for Magnetic Resonance Imaging of Colorectal Cancer with Hepatic Metastasis
Source: J Funct Biomater. 2025 Jun 20;16(7):229. doi: 10.3390/jfb16070229 (PMC12295550; doi:10.3390/jfb16070229)
Supplement: Supplementary file 1 [file jfb-16-00229-s001.zip › jfb-3647894-supplementary.pdf]

Supplemental Figures

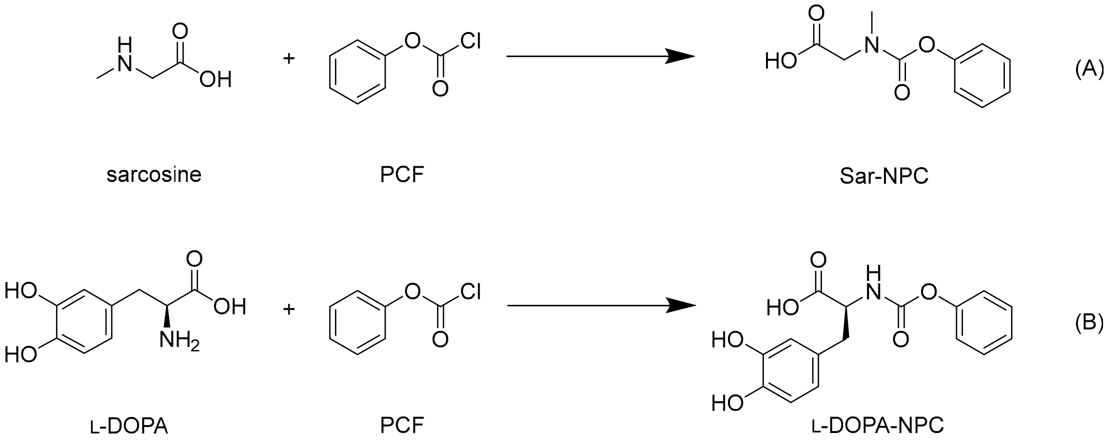

Scheme S1. Synthesis of Sar-NPC (A) and L-DOPA-NPC (B).

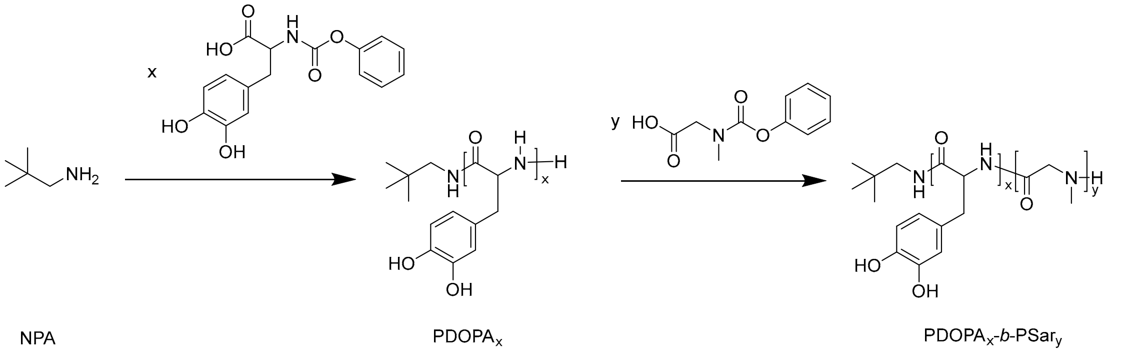

Scheme S2. Diblock copolymerization of L-DOPA-NPC with Sar-NPC.

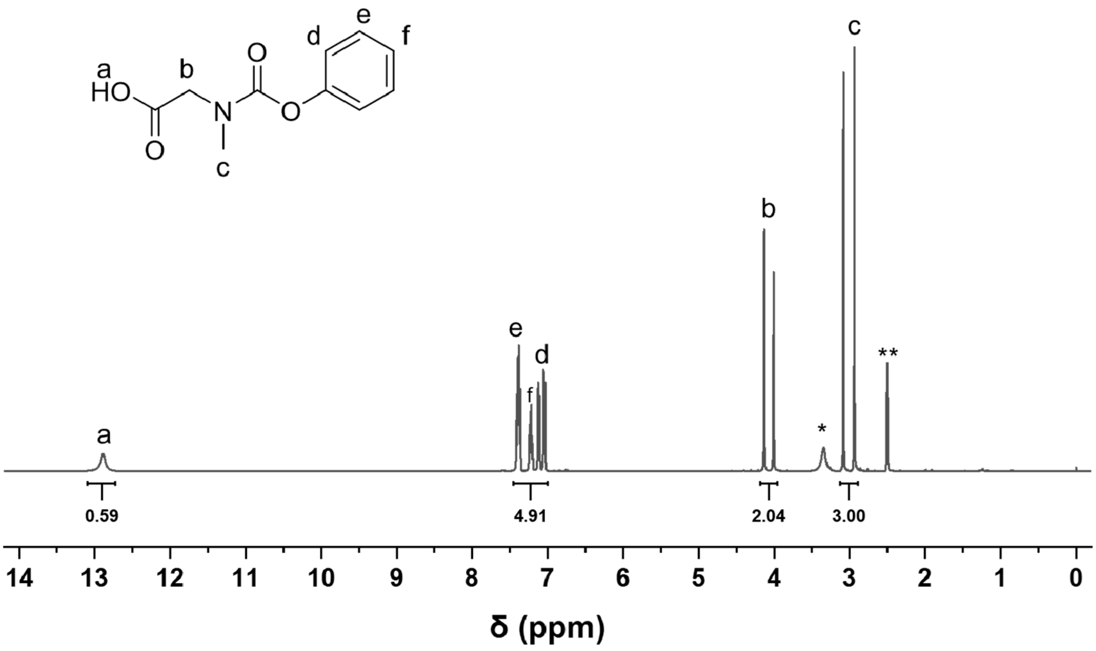

Figure S1. ^{1}H NMR spectrum of Sar-NPC in DMSO-d_6 (* H_2O, ** DMSO).

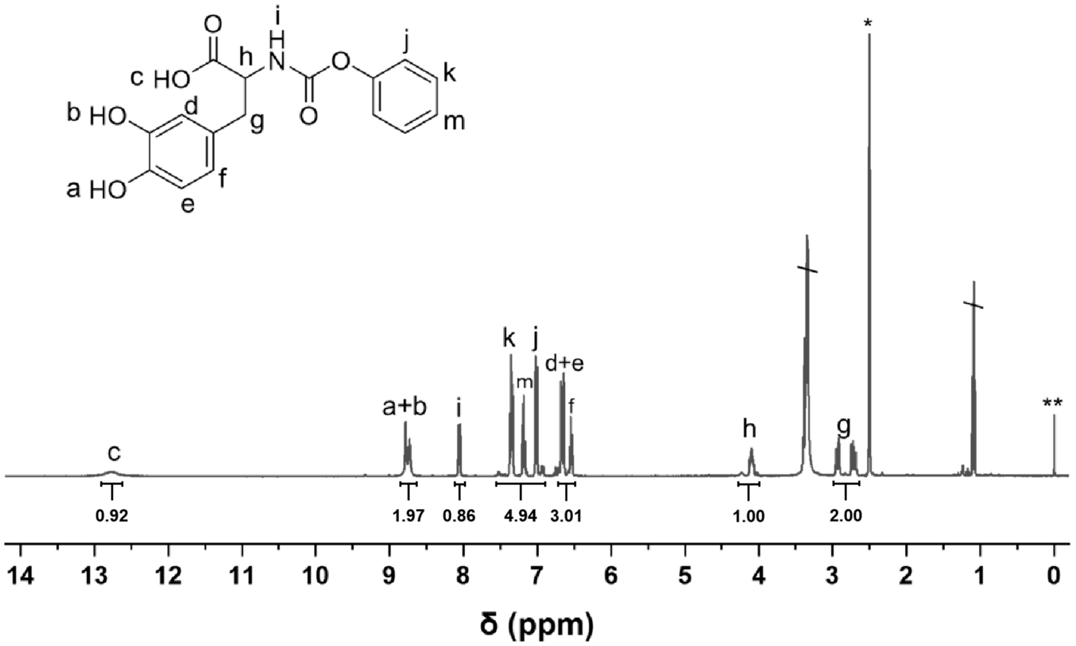

Figure S2. ^{1}H NMR spectrum of L-DOPA-NPC in DMSO-d_6 (* DMSO, ** TMS, \ diethyl ether).

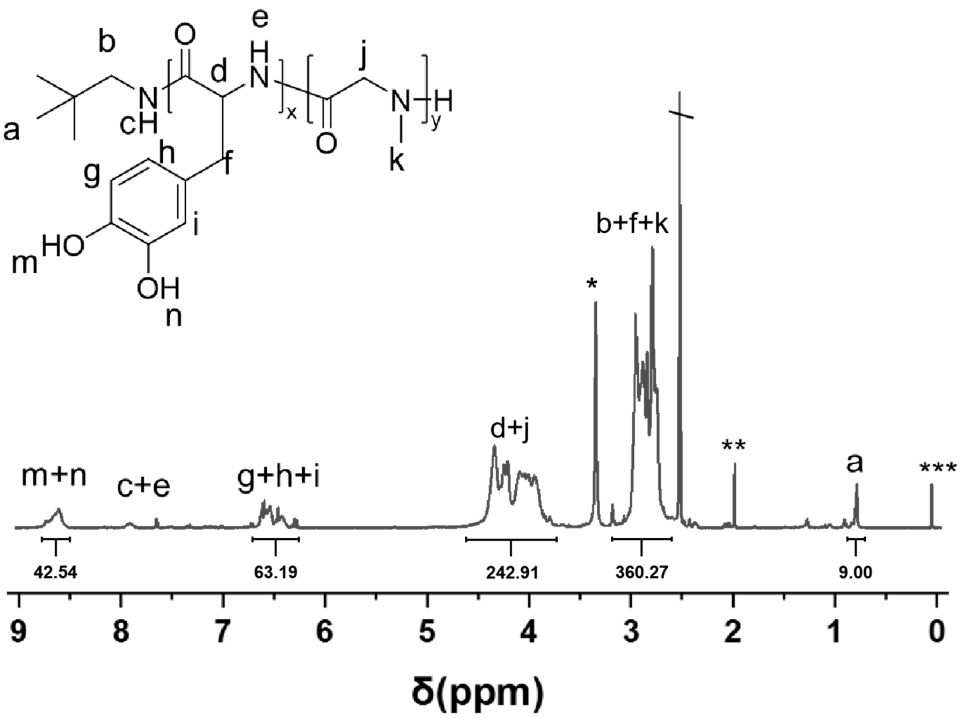

Figure S3. ^{1}H NMR spectrum of POS in DMSO-d_6 (* H_2O, ** DMAc, *** Tetramethyl silane, \ DMSO).
